# Spectral and Microscopic Behavior of Type III Femtosecond Fiber Bragg Gratings at High Temperatures

**DOI:** 10.3390/mi16030331

**Published:** 2025-03-12

**Authors:** Matilde Sosa, Maxime Cavillon, Thomas Blanchet, Matthieu Lancry, Guillaume Laffont

**Affiliations:** 1 Université Paris-Saclay, CEA, List, 91120 Palaiseau, France; matilde.sosamarti@cea.fr (M.S.); thomas.blanchet@cea.fr (T.B.); guillaume.laffont@cea.fr (G.L.); 2 Institut de Chimie Moléculaire et des Matériaux d’Orsay (ICMMO/SP2M/MAP), Université Paris-Saclay, CNRS, 91405 Orsay Cedex, France; maxime.cavillon@universite-paris-saclay.fr

**Keywords:** fiber Bragg gratings, femtosecond lasers, microvoids, high temperature

## Abstract

Fiber Bragg gratings are key components for optical fiber sensing applications in harsh environments. Microvoids, or so-called type III fiber Bragg gratings, fabricated using femtosecond lasers and the point-by-point technique, were characterized at high temperatures (>1100 °C). For this purpose, we monitored the spectral characteristics of the grating, as well as the evolution of the microstructure during a 30 min isochronal annealing process. This study allowed us to correlate the behavior of the microvoids with the spectral performances (amplitude, wavelength drift) of the sensors at very high temperatures. As the grating signal is being lost at increasing temperatures (above 1125 °C), the periodic array of microvoids becomes disordered and deformed, ultimately losing its periodic spacing.

## 1. Introduction

Monitoring and controlling industrial processes and structures in harsh environments requires sensors that are particularly adaptable to such extreme conditions, which include high temperatures, high pressures, and strong electromagnetic radiation. Optical fiber sensors are well-suited in this context given their compact size and immunity to electromagnetic interference and bring additional advantages such as resistance to corrosive environments and multiplexing capabilities. Fiber-based sensors have been recently deployed in various fields, such as aerospace [1,2], metallurgy [3,4], power generation [5,6], and nuclear reactors [7,8]. Among them, fiber Bragg gratings (FBGs), including regenerated FBGs (RFBGs), type II FBGs inscribed using femtosecond (fs) lasers (type II fs-FBGs), and type III FBGs generated using single fs pulses and the point-by-point technique (type III fs-FBGs), have proved to be ideal candidates given their particular resistance to high temperatures [9,10,11,12].

Focusing on type III fs-FBGs, there is a growing interest in their development due to their relatively simple fabrication process and flexibility in adjusting inscription parameters, thanks to the single-pulse point-by-point inscription technologies, which allow high manufacturing speed [13]. This also enables the production of sensors with spatial and spectral multiplexing [14]. This direct writing method was first presented in 2004 by Martinez et al. [15], where periodic Bragg grating patterns were created by tightly focusing single fs-laser pulses inside the fiber core while precisely translating the fiber at a constant speed along the fiber axis. In a previous study, we characterized the unitary structure of type III fs-FBGs, so-called microvoids, observing a densified shell around these voids [16]. This densified shell remains present even at elevated temperatures (up to 1100 °C), suggesting its formation under a high-pressure, high-temperature mechanism, somehow contributing to a high thermal stability compared to type II fs-FBGs. Furthermore, we found that the imprinted microvoids exhibit an ellipsoidal shape by means of high-resolution microscopic techniques [16,17]. Intriguingly, heat treatments above 1100 °C reveal that these structures begin to deform and even grow instead of being erased.

This raises the question of how the spectral characteristics of such sensors, particularly Bragg peak amplitude and its wavelength, evolve at very high temperatures in relation to microstructural changes. While previous studies have shown that at very high temperatures the Bragg peak disappears [18] and an irreversible drift in the Bragg wavelength peak occurs [19], there are no studies exploring the evolution of the microvoids during this process. In this paper, we investigate the evolution of both the spectral characteristics (such as Bragg peak amplitude and wavelength shift) and the microstructural integrity of type III fs-FBGs when exposed to extreme temperatures. Our study offers new insights into the degradation mechanisms of the grating, providing a deeper understanding of the physical changes occurring in these FBGs during high-temperature operation.

## 2. Materials and Methods

Type III fs-FBGs were fabricated at the FemtoBragg platform (CEA List, Palaiseau, France). They were inscribed using a Pharos (Light Conversion, Vilnius, Lithuania) femtosecond laser, operating at around 515 nm with a pulse duration of 170 fs. A conventional single-mode fiber SMF-28 (Corning, New York, NY, USA) is placed on the direct writing platform, after stripping its acrylate coating, and its core is aligned using a 1.4 NA, ×60 oil-immersion microscope objective. Using the point-by-point technique, the microvoids are created inside the core, focusing fs-laser single pulses while the platform moves at a constant speed along the fiber axis, according to the desired pitch (Λ) of the grating being manufactured. Fourth-order FBGs were inscribed with different parameters, i.e., changing the inscription pulse energy (between 40 nJ and 80 nJ) and the total length of the gratings (between 1.5 mm and 4 mm), so as to maintain reflectivity between 80% and 90%. Each fiber had three FBGs multiplexed in wavelength (at 1510 nm, 1540 nm, and 1570 nm), spatially separated by 1 mm and written using three different energies, as shown in Table 1.

The four fiber samples were investigated simultaneously. They were inserted into a tubular furnace (Carbolite Gero, Neuhausen, Germany) and the evolution of the reflected spectra was monitored in real time using a Tunics tunable laser and a CT400 (EXFO, Quebec, QC, Canada) with a resolution of 1 pm, as well as a splitter (4 × 1) to record simultaneously the four reflected spectra. Real-time monitoring of the furnace temperature was also performed using three thermocouples placed near the FBGs. An isochronal annealing treatment with steps of 30 min was performed continuously for 11 temperature steps ranging between 800 °C and 1175 °C (800 °C, 1000 °C, 1050 °C, 1075 °C, 1100 °C, 1112 °C, 1125 °C, 1137 °C, 1150 °C, 1162 °C and 1175 °C).

A post-treatment of the acquired signals was performed by tracking the Bragg peak of each grating using two methods: a −3 dB bandwidth method, measuring directly from the reflected spectrum without altering or processing its shape, and a third-order polynomial fit, interpolating locally the detected peak with a polynomial function. Both methods giving similar results, we opted for displaying the results using the first one. In this way, we obtained the evolution of the Bragg wavelength, as well as the evolution of the amplitude of the Bragg peak as a function of time as temperature increases.

**Figure 1:**
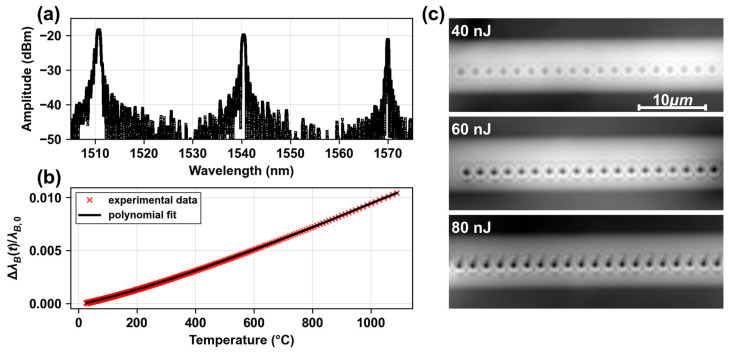
(**a**) Spectrum of power as a function of wavelength. Three FBGs are inscribed in the same optical fiber at three different wavelengths. (**b**) Wavelength variation $\frac{\Delta \lambda_{B}\left(T\right)}{\lambda_{B,0}(20\;{}^{\circ}C)}$ as a function of temperature. Red points are the experimental data (one over fifty points for visualization), and the black line the polynomial fit. Error between the fit and the experimental data is less than 3 °C. (**c**) Quantitative phase images of fourth-order FBGs using QPM (top-view of the optical fiber), for three energies of inscription: 40 nJ, 60 nJ, and 80 nJ.

In order to compare the evolution of the Bragg wavelength with the temperature measured by the thermocouple, a calibration of the gratings was performed. For this, we used a reference fiber with three gratings: at 1510 nm, with a pulse energy of 80 nJ and a length of 1.5 mm; at 1540 nm, with a pulse energy of 60 nJ and a length of 1.5 mm; and at 1570 nm, with a pulse energy of 40 nJ and a length of 4 mm. The fiber was brought to 1100 °C and then returned to room temperature in order to calibrate with data as close as possible to the maximum temperatures studied, but without losing the signal. We monitored the evolution of the Bragg peak to get the reversible wavelength shift of the grating as a function of temperature. Performing a fifth-order polynomial fit of the data, we obtained a calibration curve, where the normalized Bragg wavelength shift ($\frac{\Delta \lambda_{B}\left(t\right)}{\lambda_{B,0}}$, with $\Delta \lambda_{B}\left(t\right)=\lambda_{B}\left(t\right)-\lambda_{B,0}$ and $\lambda_{B,0}$ being the Bragg wavelength at room temperature) is translated to a temperature variation,$\;\Delta T=f\left(\frac{\Delta \lambda_{B}\left(t\right)}{\lambda_{B,0}}\right)$ with a residual error of less than 3 °C covering the entire temperature range investigated. In Figure 1a, we can see the initial spectrum of these three multiplexed gratings, and in Figure 1b, the calibration curve with experimental data (red points) and the above-mentioned five-order polynomial fit (black line). The calibration curve was constructed using data corresponding to the second peak at the resonance wavelength of 1540 nm. This selection is justified by the fact that the first part of the results focuses on the behavior of a single grating, characterized by $\lambda_{B}=1540\;nm$,$\;L_{FBG}=1.5\;mm$, and $E_{LASER}=60\;nJ$.

It is possible to observe in different studies (for instance, for type I FBGs [20], type IIA FBGs [21], regenerated FBGs [22], type II fs-FBGs [12], or sapphire fs-FBGs [23]) that the relationship between wavelength variation ($\Delta \lambda_{B}$) and temperature (ΔT) is not linear, and depends also on the specific wavelength being analyzed. However, we can make a clear distinction in sensitivity when changing the material, where sensors on sapphire fibers reach sensitivities of almost double with respect to silica-based optical fibers, for the same reference operating temperature and reference wavelength. From this calibration, we can obtain similar thermal sensitivity results to those obtained by Lerner et al. [24], where he also compares with other types of sensors in silica-based optical fibers, where the same trend is revealed. At a wavelength of reference of 1500 nm, we can measure with a sensitivity of 10 pm/°C around room temperature, 15 pm/°C around 500 °C, and 17 pm/°C around 1000 °C.

To complement the spectral measurements, we monitored the microvoid behavior by optical microscopy, with the objective of correlating it to the spectral characteristics of the FBGs during the annealing process. Following a similar thermal protocol, a sample was placed in the furnace for each of the isochronal steps of the above-mentioned process. The microvoid-based gratings were inscribed using the same parameters as mentioned before, and the fibers were glued at the ends to an Infrasil microscope slide in order to have a fixed reference to the image. After each step, the sample was taken out of the furnace to be examined under the microscope and then returned to the furnace for the next temperature step. Examination was performed using the Quantitative Phase Microscopy software technique [25] (QPM, from Iatia Vision Science, Melbourne, Australia) coupled with an optical microscope (BX60, Olympus Co., Tokyo, Japan). This technique acquires three optical images at different depths (at +1 µm/0 µm/−1 µm from the focus) using a piezo motor displacing the optical objective along its axis. A digital quantitative phase image is produced by processing the three images via solving the intensity transport equation [25]. All images are acquired with a 1.35 NA ×100 oil-immersion microscope objective (Olympus Co., Tokyo, Japan). In Figure 1c, we can observe the quantitative phase images of fourth-order FBGs for different energies of inscription: 40 nJ, 60 nJ, and 80 nJ. These images were taken at room temperature before starting the annealing process.

## 3. Results

### 3.1. Spectral Behavior of Type III fs-FBGs at High Temperatures

**Figure 2:**
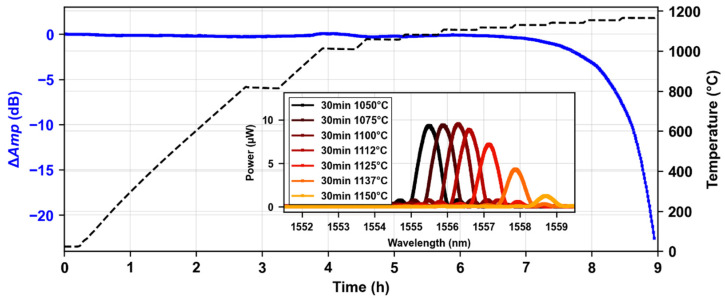
Evolution of Bragg peak amplitude over 30 min step isochronal thermal annealing up to 1162 °C. In blue (left axis), the variation of the Bragg resonance amplitude ($E_{LASER}=60\;nJ,\;\lambda_{B}=1540\;nm\;L_{FBG}=1.5\;mm$). In black (right axis), the monitoring temperature from the thermocouples. In the inset, the evolution of the Bragg peak (amplitude power, in µW, as a function of wavelength, in nm) after each temperature step between 1050 °C and 1150 °C.

In this first part of the results, we aim to show the evolution of the Bragg peak amplitude at very high temperatures in order to identify the temperature range in which it is possible to track and measure the Bragg peak wavelength. In Figure 2, we can see the evolution of the Bragg peak amplitude as a function of time over the 30 min step isochronal annealing process. In blue (left axis), one can observe the variation of the Bragg resonance amplitude for a grating that was inscribed with a 60 nJ pulse energy, being 1.5 mm long, which is similar to the one shown in Figure 1. In black (right axis), the monitored temperature from the thermocouples is provided. When one increases the temperature, the amplitude of the Bragg peak remains rather constant for temperatures below 1100 °C. However, as the annealing temperature is further increased, we can see an accelerated decay of the Bragg peak resonance, starting at the 1125 °C temperature step, leading to a complete signal loss after 30 min at 1162 °C. In the Figure 2 inset, we can observe the evolution of the Bragg peak (amplitude power, in µW, as a function of wavelength, in nm) after each temperature step between 1050 °C and 1150 °C.

### 3.2. Microstructure Behavior of Type III fs-FBGs at High Temperatures

As previously observed [16], the microvoids inscribed under conditions similar to those used for creating type III fs-FBGs (a periodic array of microvoids) evolve differently compared to nanopores at the origin of type II fs-FBGs, which are simply erased at high temperatures [26]. Instead, the microvoids first experience a reduction in size at around 1100 °C, followed by regrowth and deformation at higher temperatures. For the gratings studied here, the microvoids exhibit a similar non-monotonous behavior as the temperature increases. To investigate these changes, the 2D Fourier Transform (FT) of the images (specifically the area containing the microvoids) was computed to assess the frequency components. From the FT, the power spectrum was derived by calculating the squared magnitude of the Fourier coefficients ($P\left(u,v\right)={\left|F\left(u,v\right)\right|}^{2}$), representing the distribution of energy across different spatial frequencies (u,v). Additionally, the “spatial frequency entropy” was estimated from the normalized power spectrum ($\hat{P}\left(u,v\right)=P(u,v)/\Sigma P(u,v)$) in order to obtain a qualitative indicator of the evolution of the images as we increased the temperature, in terms of the periodicity of the grating structure and the disorder within the image. The entropy was calculated using the Shannon entropy formula [27]:

(1) $$H=-\sum_{u,v}\hat{P}\left(u,v\right)\log_{2}\left(\hat{P}\left(u,v\right)\right)$$

An increase in entropy could indicate a loss of periodicity or regular structure, which manifests in the frequency domain as a broader frequency distribution.

**Figure 3:**
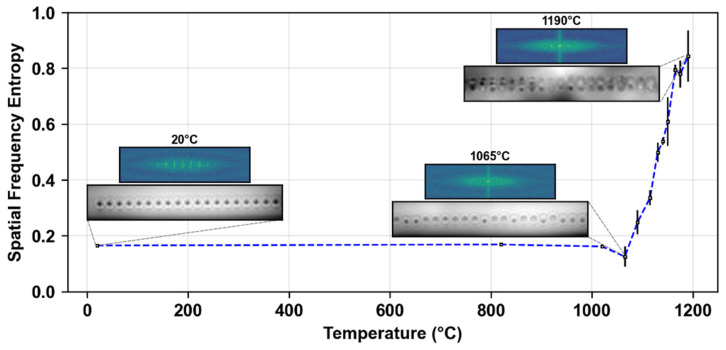
Estimation of the entropy calculated using the 2D Fourier Transform (FT) of microvoid images over a 30 min step isochronal thermal annealing process. The inset shows quantitative phase images of a fourth-order FBG ($E_{LASER}=60\;nJ$, $\lambda_{B}=1540\;nm$, $L_{FBG}=1.5\;mm$) taken at room temperature after key isochronal steps (20 °C, 1065 °C, and 1190 °C). For each QPM image, the corresponding 2D FT is displayed.

In Figure 3, we observe that the entropy increases with temperature, especially above 1100 °C. The figure also shows Bragg grating optical images at various stages of isochronal annealing (after key isochronal steps, 20 °C, 1065 °C, and 1190 °C), along with their 2D Fourier Transforms. For example, at 1065 °C, the microvoids shrink a little, and the grating initial alignment is altered. As the temperature increases beyond the glass transition temperature (approximately 1080 °C), the microvoids begin to deform and split into two or more voids, as seen in the inset of Figure 3 at 1190 °C.

We previously observed a loss of the Bragg signal at very high temperatures above 1100 °C. However, based on these images, we can see that this signal loss does not occur because the microvoid structure within the fiber optic core is erased. Instead, the microvoid structure evolves in such a way that the periodicity and regularity of the pattern gradually degrade, resulting in less reflected signal (decrease in diffraction efficiency). Eventually, the structure completely loses its periodicity; at this point, there is no longer resonance at the characteristic wavelength of the grating, and the Bragg peak can no longer be detected. To some extent, we could also infer that the loss of the signal was due to the loss of the guidance inside the optical core. However, from transmission measurements, we observed that the fiber core still guides the light at least up to 1200 °C without excessive losses (<2 dB), and still at around 1250 °C but with significant losses (>10 dB). In addition, it is also possible to observe the core-cladding refractive index contrast up to 1200 °C using QPM microscopy.

### 3.3. High Temperature Wavelength Drift Using Type III fs-FBGs

Using the previous calibration of the gratings, we calculated the reversible shift of the Bragg wavelength as a function of temperature. However, while our FBGs temperature sensor experienced long durations at very high temperatures, there was also an irreversible effect in terms of wavelength shift, also called wavelength drift. From the isochronal measurements, and using the calibration curve presented in Figure 1b, we converted Bragg wavelength evolution into temperature evolution. Comparing this temperature evolution measured with the FBGs with the temperature measured with the thermocouples, we calculated the relative temperature error as the difference between the two for each of the 30 min steps. In this way, we were able to quantify the wavelength drift mainly related to the evolution of the effective index inside the fiber core.

**Figure 4:**
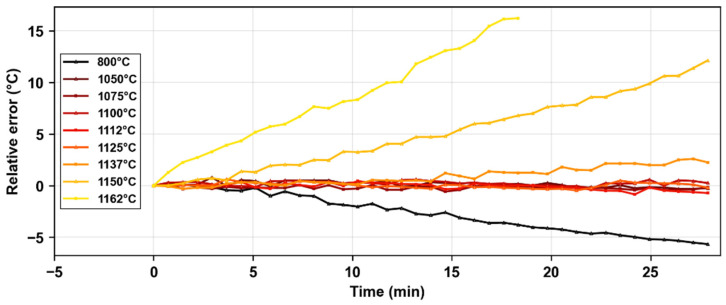
Irreversible effects of wavelength shift for each step of the isochronal thermal annealing: Relative temperature error for each step (from 800 °C to 1162 °C). From the above-mentioned calibration curve, we converted the wavelength shift into temperature, which is compared with the thermocouple one to get the relative error. ($E_{LASER}=60\;nJ$, $\lambda_{B}=1540\;nm$, $L_{FBG}=1.5\;mm$).

In Figure 4, we can observe the relative error as a function of annealing time for each of the temperature steps between 800 °C and 1162 °C. At 800 °C, we observed a negative relative error between the temperatures determined from the calibrated wavelength shift and the thermocouple ones (−0.18 °C per minute), which was probably related to a variation of the mean refractive index within the core due to stress relaxation of the fiber. For higher temperatures up to 1125 °C, there was almost no error between both measurements indicating no significant drift on this timescale. However, as we increased the temperature even more (to temperatures above 1137 °C), the relative error started to get progressively higher (0.05 °C per minute at 1137 °C, 0.5 °C per minute at 1150 °C, and 0.9 °C per minute at 1162 °C) until the erasure of the peak during the 1162 °C step. In the discussion section, we will analyze in detail the different contributions to wavelength drift and the most likely phenomena occurring in each case.

**Figure 5:**
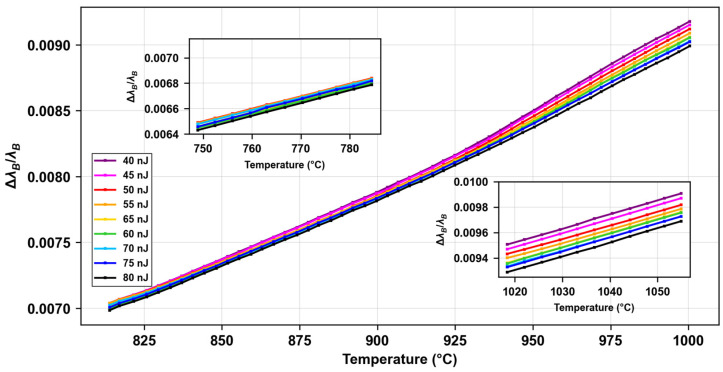
Normalized wavelength shift as a function of temperature, as we increase temperature from step at 800 °C to the next one at 1000 °C, for FBGs with different energies of inscription (between 40 nJ and 80 nJ). Left inset normalized wavelength shift as a function of temperature for in a range varying from 750 °C to 790 °C. Right inset for temperatures ranging from 1020 °C to 1060 °C. For sake of comparison, *x* and *y* axes in both insets have the same scale.

For the same experiment but considering specifically the wavelength shift during the increase of the temperature from 800 °C to 1000 °C, we observe that the normalized wavelength shift depends on the energy of inscription at very high temperatures (i.e., above 900 °C). In Figure 5, we observe the evolution of the normalized wavelength shift as a function of temperature as we increase the temperature from the step at 800 °C and the next one at 1000 °C. We can see the different curves that start to separate at temperatures between 925 °C and 950 °C, and a *rainbow effect*, where the nine curves are arranged from the highest energy (in black, 80 nJ) to the lowest (in purple, 40 nJ), so that there is a larger shift for smaller inscription energies. As we further increase the temperature (above 1000 °C), the slope of these curves hardly changes, so they have the same sensitivity but slightly different ordinates at the origin. We can see this clearly in the inset at the bottom right, where normalized wavelength shift is plotted as a function of temperature, in the range between 1020 °C and 1060 °C, where temperature is increasing between two consecutive steps. To complement the analysis, we can see in the inset on the top left that even at quite high temperatures (between 750 °C and 790 °C) this effect is not yet visible, and all curves are close, which is also the case for temperatures below this range. Notice that the *x* and *y* axes of the insets all have the same scale in order to compare the different plots.

## 4. Discussion

In the first part of the results, we observed how the erasure of the Bragg peak is related to microvoid deformation inside the optical fiber core. Unlike nanogratings [26,28], these microvoids are not erased from the silica glass, but first they lose their periodicity. This raises the question of what causes such deformation in these thermal conditions. By modeling the problem using the Rayleigh–Plesset (RP) equation, which initially describes the dynamics of a spherical bubble in an incompressible fluid and incorporates parameters such as viscosity, surface tension, and pressure difference, we can hypothesize about the potential causes of this deformation. In its simplified form, the RP Equation (2) takes the following form,

(2) $$\frac{dR}{dt}=\frac{R\Delta P}{4\eta }-\frac{\sigma }{2\eta },$$

where dR/dt is the evolution of the void radius (R, in m) as a function of time (t, in s), ΔP is the pressure difference between inside and outside the void (in Pa), σ is the surface tension (in $J/m^{2}$), and η the glass viscosity (in Pa × s).

**Figure 6:**
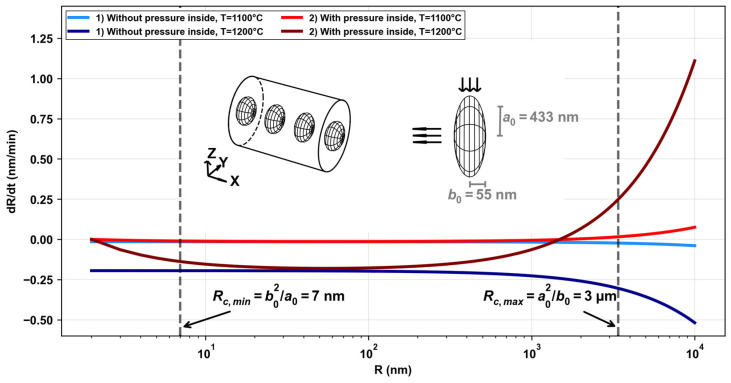
Simplified Rayleigh–Plesset equation, dR/dt (in nm/min) as a function of R (in nm and in logarithmic scale), for two different cases, with and without pressure inside the microvoid (in red and blue tones, respectively). For each case, there are two plots for two different temperatures (1100 °C and 1200 °C). Left inset is a model of some voids inside the optical fiber core for visualization of the problem. Right inset, one void with its dimensions. The arrows indicate the possible growth and shrinkage of the void at high temperatures.

If we model the issue similarly to how nanopores or nanogratings are handled [26,28], considering that the microvoid is effectively empty (or with a negligible amount of gas inside), microvoids would be simply erased at a certain temperature depending on their initial radius. In these conditions, even with arbitrary large radii, dR/dt is always negative, mostly constant (=−σ/(2η)) up to considerably large radii, around 1 µm. The temperature of the surrounding glass, and hence the viscosity, dictates the erasure rate. Such modeling for two different temperatures (1100 °C and 1200 °C) are reported in Figure 6 (blue tones), showing dR/dt (in nm/min for convenience), for a large range of radii (R, in nm).

The equation is derived under the assumption that the microvoid is spherical throughout its expansion and collapse. This spherical symmetry simplifies the mathematical modeling, since it guarantees that the radius and the acting forces are uniform in all directions. However, an important factor to consider is that they are not spherical but rather take the form of an oblate spheroid [16]. This is schematically illustrated in Figure 6. This alters the erasure rate of the radius, as we must account for the radius of curvature. To do this, a more complete model incorporating the geometrical characteristics of an oblate spheroid must be developed. While this is beyond the scope of this study, we chose a simplified approach to get the main physical effects resulting from this shape difference. We will focus on what happens with the limiting cases of the main radii of curvature (maximum value at $a_{0}^{2}/b_{0}$ and minimum value at $b_{0}^{2}/a_{0}$), with $a_{0}$ and $b_{0}$ being the semi-major axes as shown in Figure 6. We also consider that some gas can potentially fill the cavity during the thermal treatment. Using an ideal gas law, valid for low to moderate pressures, the pressure difference between inside and outside the microvoid will depend on the volume and temperature ratio relative to the initial and ambient conditions. In this case, a simplified RP equation will develop as follows:

(3) $$\frac{dR}{dt}=\frac{RP_{0}}{4\eta \left(T\right)}\left(\frac{TV_{init}}{T_{init}V}-1\right)-\frac{\sigma_{0}}{2\eta \left(T\right)}\left(1-\frac{2\delta }{R}\right),$$

where V, T, $V_{init}$, and $T_{init}$ all correspond, respectively, to the volume and temperature of the void at a given time (t) and temperature (T) during the heat treatment, and the initial volume and temperature when heat treatment started. Here, the surface tension dependence on the curvature radius is introduced through the well-known expression $\sigma =\sigma_{0}\left(1-\frac{2\delta }{R}\right)$, with $\sigma_{0}=0.3\;J/m^{2}$ and δ being the Tolman length (taken to be about 1 nm). When introducing a pressure inside the void, dR/dt will become positive for some values of R<2δ, that is, 2 nm. This situation is not reached. However, it gives a physical representation of what would happen if a bubble containing gas collapsed, when R → 2δ, dR/dt → 0, finding an equilibrium condition. For further analysis, we will consider the case where volume remains constant ($V_{init}=V)$, which is quite in agreement with our observations, and where there is an initial pressure outside the microvoid $(P_{0}$) of one atm ($\approx 10^{5}\;Pa)$.

In addition, we consider the possibility of gas migration, oxygen in particular, at high temperatures (>800 °C), which is consistent with thermally annealed SiO_2_ that absorbs O_2_ from the ambient atmosphere [29]. In particular, the characteristic length of diffusion at 1175 °C and for a duration of 30 min is in the order of magnitude of the optical fiber radius (62.5 µm), which is within the order of magnitude of the problem analyzed in this discussion. Therefore, we supposed that beyond a certain temperature, the situation of a filled void (becoming thus “a bubble”) is realistic.

In Figure 6, we compute dR/dt as a function of R, for different cases using Equation (3) (red tones). In some particular situations, the voids can regrow at high temperatures (the left term on the right side predominates over the right term). We can also see, in the dotted line, the limiting values of the radii of curvature for the experimentally observed voids. For a very small radius of curvature, as is the limiting case, dR/dt is negative (the void collapses), while for a large radius of curvature, dR/dt is positive, i.e., the void grows. This effect is more pronounced at higher temperatures. This could explain the regrowth observed under QPM and optical microscopy. If we considered a spheroidal shape, the conditions to start observing a regrowth should require voids typically twice as large as what is experimentally observed, reinforcing the idea that a spheroidal shape with “flat edges” would facilitate the regrowth of the void during thermal treatment. As a final note, one should bear in mind that the ellipsoid filled with a gas would ultimately evolve towards a spherical shape with a radius defined by the Young–Laplace equation ($R_{\infty }=2\sigma /\Delta P$) before it is ultimately erased. 

The evolution of the microstructure at high temperatures also results in a wavelength drift as seen in Figure 4. Based on the relative temperature error for each temperature step, this shift can be mainly attributed to variations in the effective index of LP_01_ mode propagation within the optical fiber core. For instance, at 800 °C, we observe a negative wavelength shift (blue shift), indicating a decrease in the mean refractive index. A possible explanation for this is the relaxation of residual stress, which originates from the fiber manufacturing process. This stress is due to the mismatch of viscoelastic properties of the core and cladding (mechanical stress induced during drawing) and the mismatch of the thermal expansion coefficients between the core and cladding, which places the core under compression. Once a sufficiently high temperature is applied (around $0.8{\;T}_{g}$, being $T_{g}≅1080\;{}^{\circ}C$ the glass transition temperature of SMF-28 fiber) this compressive stress relaxes, leading to a negative change in the refractive index.

At higher temperatures (between 1137 °C and 1162 °C), we observe a red shift in the wavelength. If we consider the possible contributions that make the mean refractive index change at high temperatures, we would have to consider first the evolution of the microvoids in the core of the optical fiber. As we observed in our experiments, the voids at these temperatures deform and even increase in size. Therefore, this array of microvoids would have a larger contribution to the overlap integral of the propagation mode and would reduce the mean refractive index and hence the Bragg wavelength shift.

Alternatively, we could examine what happens to the silica glass structure as we increase the temperature. Fictive temperature is the temperature at which the glass structure freezes during cooling, reflecting its structural state as if it were in equilibrium at that temperature [30]. Annealing at high temperatures allows the glass structure to reach a new equilibrium, restoring the fictive temperature to the annealing temperature and influencing the physical properties of the glass, such as its density and refractive index. Particularly, for silica glasses, the refractive index increases as a function of the fictive temperature in the temperature range of interest (between 1000 °C and 1200 °C) [31]. There is a minimum heat treatment time at a particular temperature to ensure that a structural equilibrium is established. This relaxation time is given by the ratio between the viscosity of the material as a function of temperature η(T) and the shear modulus G. The higher the heat treatment temperature, the shorter the relaxation time is. In particular, the relaxation time has an exponential decay, being approximately 216 days at 800 °C, 8 h at 1000 °C, and only 30 min at 1100 °C for the GeO_2_-SiO_2_ core composition of SMF-28 fiber. Thus, taking into account our conditions, i.e., 30 min step annealing above 1100 °C, this is a suitable hypothesis that would explain the observed red shift of Bragg wavelength over such high-temperature annealing.

Additionally, we observed an effect of the inscription energy on the evolution of the normalized Bragg wavelength as a function of time and temperature, starting at around 900 °C. This could be due to a change in the refractive index caused by a local mechanism related to the microvoid. We suggest that the size of the void, and consequently the mode overlap integral around the void, may be involved. Another more plausible explanation is related to the relaxation of the densified shell around the voids. Microvoids, particularly, are considered to be formed under a high-pressure, high-temperature mechanism, given the fs pulse energy and tight focusing conditions. Getting into the detail of the densification relaxation process, it will be different depending on the thermo-mechanical path [32]. For instance, densification through compression at high temperatures will relax slower or at higher temperatures, leading to a more stable and more homogeneous sample [33]. If we take the example of three compression parameters studied (5 GPa–1020 °C, 5 GPa–750 °C, and 5 GPa–420 °C), we can see that relaxation times vary from 155 min, 51 min, and 7 min, respectively, for an annealing temperature of 850 °C. Particularly, in the case with a higher temperature, we can see that at an annealing temperature of 900 °C the relaxation time is 33 min, i.e., a condition similar to our experiments. Therefore, relaxation of the microvoid shell is a possible explanation of the observed shifts. Following that view, the permanent index variation within the shell is of positive sign, and when relaxed it would create a blue shift in the Bragg wavelength, being more significant for higher inscription energy.

## 5. Conclusions

In conclusion, fs-type III FBGs, fabricated using the point-by-point technique, were characterized at elevated temperatures up to 1190 °C. We observed a degradation in the reflected peak amplitude of the grating at temperatures above 1125 °C, and how this degradation correlates with alterations in the microstructure of the voids. As the temperature rises, the periodic array of microvoids becomes misaligned, deformed, and loses its periodicity, leading to the observed decay of the Bragg reflectivity. The microvoid deformation was discussed through a modified Rayleigh–Plesset model considering the presence of O_2_ within the voids and their oblate shape resulting in a surface tension that depends on the curvature radius. Several hypotheses were proposed to try to explain the physical phenomena contributing to the observed wavelength drift, such as redshift identified at temperatures above 1125 °C, possibly related to a structural relaxation of the glass above the glass transition temperature. Further research will be required to explore in greater detail the effects of prolonged high-temperature and related wavelength drift, with the goal of enhancing the reliability and predicting the lifetime of these sensors in hostile environments.

## Figures and Tables

**Table 1:** Fiber samples with all inscription parameters.

Fiber Sample	$\lambda_{B}\;(nm)$	$L_{FBG}\;(mm)$	$E_{LASER}\;(nJ)$	Reflectivity (%)
1	1510	1.5	75	86
	1540	1.5	65	84
	1570	3.0	45	89
2	1510	1.5	80	89
	1540	1.5	60	87
	1570	4.0	40	87
3	1510	1.5	70	87
	1540	2.0	55	90
	1570	2.5	50	89
4	1510	1.5	70	87
	1540	2.0	55	90
	1570	2.5	50	82

## Data Availability

Data underlying these results may be obtained from the authors upon reasonable request.
